# Effects of grape and pomegranate waste extracts on poultry carcasses microbial, chemical, and sensory attributes in slaughterhouse

**DOI:** 10.1002/fsn3.1840

**Published:** 2020-09-04

**Authors:** Majid Javanmard Dakheli

**Affiliations:** ^1^ Food Technologies Group Department of Chemical Engineering Iranian Research Organization for Science and Technology (IROST) Tehran Iran

**Keywords:** decontamination, grape, pomegranate, poultry, slaughterhouse, waste

## Abstract

Contamination of poultry carcasses is considered as a critical point in the evaluation of poultry meat safety. The present study aimed at determining the decontamination effects of natural antimicrobial derived from grape waste extract and pomegranate waste extract (GWE and PWE) on poultry carcasses in a slaughterhouse. Poultry carcasses were treated in chiller with concentrations of 0, 2, 4 and 6% of pomegranate and grape waste extracts. Pomegranate and grape waste extracts contained 432.20 and 328.43 mg GAE/g total phenolic compounds. These extracts showed significant antimicrobial effect on the main poultry bacteria in vitro. On the first day of cold storage, significant reduction in total bacterial counts (*p* < .05) was observed in treated carcasses. After 3 days of storage time, total bacteria, *Staphylococcus aureus*, and *Escherichia coli* reduced significantly (*p* < .05) compared to untreated samples. At sixth and ninth days of storage time, significant reduction in total volatile nitrogen (TVN), total bacteria counts, *Staphylococcus aureus*, coliforms, and *Escherichia coli* were observed. Sensory attributes in treated carcasses with PWE and GWE have been enhanced significantly compared to untreated during acceptable shelf time (*p* < .05). Based on the results, pomegranate and grape waste extracts can be used to preserve and improve the shelf life of the poultry carcasses close to the standard range until the ninth day of storage. Application of pomegranate and grape waste extracts in slaughterhouse could be considered as an environmentally, natural and safe decontamination intervention in integral food safety system.

## Graphical abstract

Decontamination with natural antimicrobials extracted from pomegranate and grape waste extract treatment effectively reduced aerobic bacteria in carcasses 2.00 – 2.22 log CFU/g. No negative sensory changes. Practical and affordable. Can easily be implemented in slaughterhouses.

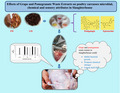

## 1 INTRODUCTION

In recent years, in response to consumers' concerns around synthetic preservatives, considerable effort has been made to find novel antimicrobials derived from a variety of natural sources. In general terms, food preservative refers to artificial or natural additives for inhibiting bacterial and fungal growth in order to improve the quality and shelf life of food products. Natural antimicrobials can be obtained from different sources including plants, animals, bacteria, algae, and fungi.

The application of natural antimicrobials recently gained interest in the food industry. This intervention in poultry slaughterhouse and processing is safe, easy to handle, and environmentally friendly. Also, many companies in the meat industry have started to use more natural antimicrobials rather than synthetic antimicrobial chemicals in an effort to have a “clean label” on their products.

Consequently, there is ongoing research to find a natural reservoir of phytochemical compounds that could be used as an alternative to antibiotics and synthetic chemicals, which could cause serious side effects. While the cost and availability are always the main issues for manufacturers, by‐products of processed foods have received great attention as potential sources of low‐cost antibacterial compounds (Samant et al., 2015).

Poultry slaughtering required applicable and safety methods to reduce bacterial loads in poultry carcasses drastically. Decontamination methods are chemical, physical, a combination of two, and biological (Zweifel & Stephan, 2014). The decontamination of poultry carcasses is of worldwide interest. Physical interventions and chemical treatments are frequently used for the removal of surface contamination from poultry carcasses. Among the physical interventions used for the decontamination of poultry carcasses, water‐based treatments (spraying, immersion, immersion/spray chilling) predominate. Chemical interventions used for the decontamination of poultry carcasses primarily comprise organic acids (in particular lactic acid), chlorine‐based treatments, or phosphate‐based treatments (in particular TSP). In addition, a wide variety of other substances and combinations has occasionally been tested. Biological interventions such as bacteriophages and bacteriocins (e.g., nisin) show promise as novel carcass interventions (Zweifel & Stephan, 2014). Decontamination of poultry carcasses by different treatments was reviewed (Loretz, Stephan, & Zweifel, 2010).

Oftentimes, large amount of by‐products in food industry, including fruit pomace, seeds, peels, pulps, unused flesh, and husks have been considered as waste, while recent studies revealed that they are promising sources of bioactive agents and valuable components with several functionalities (Gaber, Abd‐Ella, Abou‐Elhagag, & Abdel‐Rahman, 2015; Mamma, Kourtoglou, & Christakopoulos, 2008; Nitschke & Costa, 2007). It has been also confirmed that these unusable parts have a similar or even higher proportion of bioactive compounds than the usable parts, for example, the total phenolics in peels of lemons, oranges, and grapefruits have been found to be 15% higher than the level found in the peeled fruits (Ramadan et al., 2015). Accordingly, they also have a wide range of antimicrobial properties due to the total contains phenolic compounds (polyphenols, tannins, and flavonoids).

Pomegranate (*Punica granatum* L.) is the fruit of a deciduous shrub that is native of Mediterranean and Southern Asia. Iran is one of the largest producers of pomegranate by producing around 1.09 MT in a year under the local name of Anar (https://www.maj.ir, 2017). This fruit has a long history in traditional medicine, for centuries; different parts of pomegranate like bark, leaves, immature fruits, and fruit rind have been used for traditional treatment of diseases like dysentery diarrhea, helminthiasis, and respiratory pathologies (Braga et al., 2005; Choi et al., 2011; Jayaprakasha, Rao, & Sakariah, 2006; Sánchez‐Lamar et al., 2008). Phenolic compounds of pomegranate have high biological activities such as antioxidant, antimicrobial, antimutagenic, and anticarcinogenic activity (Gullon, Pintado, Pérez‐Álvarez, & Viuda‐Martos, 2016; Mansour et al., 2013; Sadeghi, Jannat, Oveisi, Hajimahmoodi, & Photovat, 2009). Gallic acid, ellagic acid, and punicalagin are important pomegranate peel polyphenols (Akhavan, Barzegar, Weidlich, & Zimmermann, 2015; Qu, Breksa, Pan, & Ma, 2012).

Grape (*Vitis* spp.) is one of the fruit cultivated almost in all regions of the world (Gruenwald, Brendler, & Jaenicke, 2007). It is a common commodity in forms of grape juice, jams, and raisins in the food market all over the world. Numerous studies focused on the health‐promoting and antioxidant effects of grapes (Adámez, Samino, Sánchez, & González‐Gómez, 2012; Baydar, Sagdic, Ozkan, & Cetin, 2006; Jayaprakasha, Selvi, & Sakariah, 2003). These studies confirmed various biological capacities such as antioxidant and antibacterial for this plant. Grapes are also well known for the high level of polyphenol content, which is a novel antimicrobial agent (Abtahi, Ghazavi, & Karimi, 2011; Rhodes, Mitchell, Wilson, & Melton, 2006). Previous studies mostly explored the antimicrobial activity of pomegranate and grape based on extracts from different parts of these fruits separately (Adámez et al., 2012; Al‐Zoreky, 2009; Gould, Fielder, Kelly, & Naughton, 2009; Machado, Santos, Correia, & Carvalho, 2003).

The present study aimed to determine the effects of natural extracts derived from grape and pomegranate waste on the decontamination of poultry carcasses as a novel treatment in a slaughterhouse.

## 2 MATERIAL AND METHODS

### 2.1 Preparation of pomegranate waste extract (PWE) and grape waste extract (GWE)

*Pomegranate fruits* were purchased from the local market in Tehran, and red grape pomace was donated by Alifard Suichch co (Saveh, Iran). Fruit pomace was washed and was dried at 38°C for about 2 days. In the next steps, *the dried* samples were *ground* separately with an electric grinder (Moulinex, Spain) *into powder* and then sieved through a fine‐mesh metallic sieve and stored in a refrigerator until the next stage. 20 g of fine powders from *pomegranate* and grape waste was weighed. These two samples (20 g) were separately dissolved in 200 ml sterile distilled water and then maintained overnight at 25 ± 1°C in laboratory conditions for 24 hr. The obtained extracts were filtered through double‐layer gauze. Subsequently, the filtered extracts were centrifuged at 5,000 rpm for 15 min. All obtained supernatants were placed in Petri dishes (10 mm), then frozen at −20°C, and finally lyophilized at −55°C under vacuum (Ilshin Lab. Co., Ltd., Yangju‐si, Korea) for 48 hr.

### 2.2 Treatment

Poultry carcasses were randomly selected during the poultry slaughter process at a commercial type slaughterhouse in the Tehran suburb area (Kushan co.). The carcasses selected uniformly and gathered into three groups for treatment application as described below compared with control. The samples were collected after the last chiller which carcasses be holding there for 5 min before packaging. Treatments and controls were composed of three carcasses. Treated samples immersed 5 min in chiller water containing 2%, 4%, and 6% (W/V) of pomegranate and grape waste extracts.

### 2.3 Chemical composition of the extracts

The total phenolic content of crude extract was determined by Folin‐Ciocalteu method (Singleton et al., 1999).

Polyphenol analyses of the liquid extracts and lyophilized dried powder from prepared from PW and GW were performed by high‐performance liquid chromatography (HPLC) based on Cheaib et al. (2018) method.

### 2.4 In vitro antibacterial activity assay

The two bacteria were provided by the Persian Type Culture Collection (PTCC) Iranian Research Organization for Science and Technology (Tehran, Iran). They were one Gram‐positive bacterium (*Staphylococcus aureus* PTCC 1399) and one Gram‐negative bacterium (*Escherichia coli* PTCC 1431). They were stored in 50% glycerol (v⁄v) at −20°C for 24 hr before starting the experiment, and the frozen bacteria were grown overnight in Mueller Hinton Broth (25 ml) at 37°C. After spending 24 hr, the overnight cultures of *E. coli* and *S. aureus* were centrifuged (3,146 *g*‐force) and then the supernatant was *discarded*. *Bacterial pellets* were resuspended in sterile normal saline and adjusted to 0.5 MacFarland standards (1.5×10^8^ CFU/ml), prepared by adding 0.05 ml of barium chloride (BaCl_2_) (1.17% BaCl_2_.2H_2_O) to 9.95ml of H_2_SO_4_ (1%) with constant stirring (NCCLS, 2000).

The MIC of the PWE and GWE extracts was determined as the lowest concentration that completely inhibited bacterial growth after 48 hr of incubation at 37°C. Positive and negative cultures were also prepared.

### 2.5 Microbial analysis of poultry carcasses

Microbial analysis of aerobic mesophilic bacteria, coliform count, *Salmonella* spp., and *E. coli* was carried out on the day of arrival and months 3, 6, and 9 of storage for each sample using the procedure by ISIRI (No: 9263 and 5272). For mesophilic aerobic plate counts, 25 g of samples from each chicken meat were cut out aseptically with a sterile pastry cutter and bistoury, diluted 1:10, and blended for 2 min with 225 ml of sterile peptone water with a blender. Subsequent dilutions were prepared by mixing a 1‐ml sample with 9 ml of sterile peptone water. For bacterial counts, volumes of 0.1 ml of selected dilutions were spread, in duplicate, on plates containing solid nutrient agar (Merck), and incubated for 24–48 hr at 35°C. Microbial counts were expressed as the number of viable bacterial colonies per gram (log CFU/g). For coliform counts, volumes of 1 ml of selected dilutions were spread, in duplicated onto plates containing approximately 12 ml of melted Violet Red Bile Agar (VRBA), and incubated for 48 hr at 37°C. For isolation of *Salmonella* spp., 25 g of chicken meat was incubated at 37°C in 225 ml of Lactose Broth for pre‐enrichment. Selective enrichment was done in Selenite Cystine Broth at 37°C and Tetrathionate Broth at 44°C followed plating on Brilliant Green Agar and Bismuth Sulfite Agar plates for detection of characteristics colonies of *Salmonella* after incubation for 48 hr.

### 2.6 Total volatile nitrogen

Total volatile nitrogen (TVN) was determined as described by Mwansyemela (1973).

### 2.7 Sensory analysis

For sensory testing, samples were evaluated by a 10 panelist‐trained taste panel consisting of students and scientific staff in the Iranian Research Organization for Science & Technology. The taste panelists were trained to familiarize fresh poultry fillets with common sensory attributes, particularly poultry attributes. The description of each attribute was developed and clearly understood among the trained panelists. Samples from control were evaluated as compared with treated samples for differences in appearance, texture, color, taste, and general acceptance. When differences were found, the degree of difference was measured by a modified method from a 5‐point rating scale. The products were shallow fried before serving (Kanatt, Chander, & Sharma, 2010).

### 2.8 Statistical analysis

Microbiological data were transformed logarithmically before statistical analysis. Means for each treatment were analyzed by analysis of variance (ANOVA) procedure of SPSS 11.5 for Windows. Least square means were separated when the treatment effect was significant in the ANOVA table (*p* < .05).

## 3 RESULTS AND DISCUSSION

### 3.1 Chemical composition of the extracts

**FIGURE 1:**
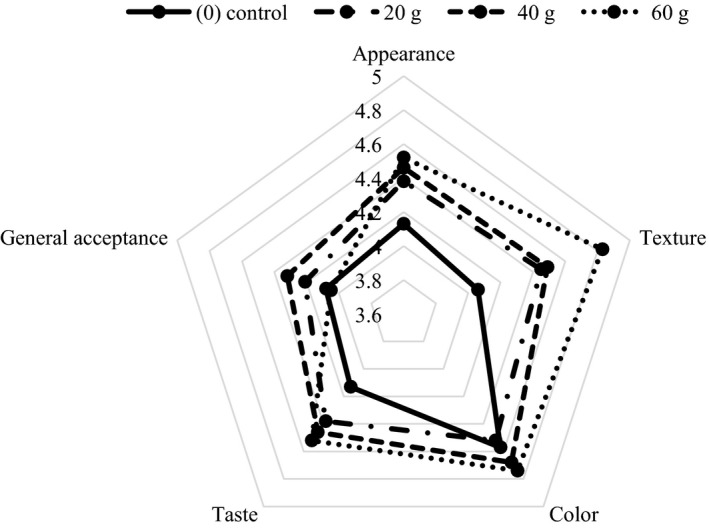
*Sensory evaluation of poultry carcasses containing pomegranate extract during storage at 6 days*

Total phenolic compounds of PWE and GWE were 432.20 and 328.43 mg GAE/g, respectively. The main polyphenols of PWE and GWE were shown in Table 1. The main polyphenols in PWE were punicalagin A (2.37 mg/g) and punicalagin B (1.48 mg/g), and ellagic acid (0.274 mg/g; Figure 1), and in GWE were rutin (1.23 mg/g), catechin hydrate (1.38 mg/g), epicatechin gallate (1.12 mg/g), gallate acid (1.32 mg/g), isorhamnetin (0.82 mg/g), ferulic acid (0.58 mg/g), and caffeic acid (0.23 mg/g).

**TABLE 1:** Pomegranate waste and grape waste extract major phenolic composition (mg/g)

Grape waste								Pomegranate waste		
Quercetin	Rutin	Catechin Hydrate	Epicatechin Gallate	Gallic acid	Ferulic acid	Caffeic acid	Isorhamnetin	Ellagic acid	Punicalagin A	Punicalagin B
1.17	1.21	1.38	1.12	1.32	0.58	0.23	0.82	0.274	1.484	2.375

### 3.2 In vitro antimicrobial effects of PWE and GWE

The results demonstrated in Table 2 indicate that PWE and GWE presented antimicrobial activity against *S. Typhimurium, E. Coli, and S. aureus* with minimum inhibitory concentration (MIC).

**TABLE 2:** Antibacterial effect of pomegranate waste and grape waste extract on the main poultry carcasses pathogenic bacteria by macro dilution assay (mm)

	Extraction time (hr)	Ethanol/Water ratio	Inhibition zone (mm)*		
			*S. typhimurium*	*E. Coli*	*S. aureus*
Grape waste	24	40/60	8.00 ± 0.89^*a^	8.83 ± 0.67^a^	10.08 ± 0.16^a^
		60/40	9.45 ± 0.24^b^	10.50 ± 0.37^b^	12.22 ± 0.46^b^
		80/20	8.34 ± 0.48^a^	9.50 ± 0.77^a^	11.22 ± 0.34^a^
Pomegranate waste	24	40/60	11.66 ± 0.89^a^	11.00 ± 1.00^a^	15.33 ± 0.57^a^
		60/40	13.00 ± 0.16^b**^	12.00 ± 1.00^b^	18.00 ± 1.00^b^
		80/20	11.16 ± 0.23^a^	11.09 ± 0.44^b^	13.66 ± 0.57^c^

* Mean ± *SD*

** Unsubscribed letters in each column indicate a significant difference at the 5% level

### 3.3 Antimicrobial effects of PWE and GWE on poultry carcasses

The results of the total counts of microorganisms, coliforms, *E. coli*, *S. Aureus,* and *Salmonella Typhimurium* from different samples of poultry meat during storage time in 1, 3, 6, and 9 days at 4°C are shown in Tables 3.

**TABLE 3:** Microbial loads of poultry carcasses treated with pomegranate and grape waste extract (immersed 5 min in chiller)

Storage day	Extract/Chiller water (ml/L)		PH	*S. typhimurium*	*S. aureus* (Log CFU/g)	*E. Coli* (Log CFU/g)	Coliforms (Log CFU/g)	Total Bacteria (Log CFU/g)
1	GWE	20	5.1	+	2.00 ± 0.02^a^	1.63 ± 0.06^a^	1.95 ± 0.10^a^	2.62 ± 0.05^a^
		40	5.2	−	–	1.54 ± 0.12^a^	1.68 ± 0.00^a^	2.48 ± 0.15^a^
		60	5.8	−	–	1.48 ± 0.10^a^	1.75 ± 0.03^a^	2.36 ± 0.08^b^
	PWE	20	4.9	+	–	1.46 ± 0.06^a^	1.88 ± 0.11^a^	2.69 ± 0.03^a^
		40	5.4	−	–	1.46 ± 0.01^a^	1.60 ± 0.08^a^	2.42 ± 0.04^a^
		60	5.1	−	–	1.36 ± 0.07^a^	1.60 ± 0.12^a^	2.23 ± 0.08^b^
	Control		5.7	+	2.00 ± 0.06^a^	1.62 ± 0.03^a^	1.70 ± 0.23^a^	3.11 ± 0.15^c^
3	GWE	20	6.52	+	3.00 ± 0.02^a^	3.69 ± 0.06^a^	4.34 ± 0.03^a^	6.81 ± 0.10^a^
		40	6.14	−	2.90 ± 0.07^a^	3.47 ± 0.04^a^	4.44 ± 0.03^a^	6.85 ± 0.04^a^
		60	6.49	−	‐	3.60 ± 0.00^a^	4.16 ± 0.014^a^	5.69 ± 0.06^b^
	PWE	20	6.14	+	‐	3.24 ± 0.01^a^	4.28 ± 0.01^a^	5.79 ± 0.03^b^
		40	6.47	−	‐	3.35 ± 0.03^a^	4.22 ± 0.05^a^	5.71 ± 0.00^b^
		60	6.32	−	‐	3.19 ± 0.04^a^	4.06 ± 0.03^a^	5.30 ± 0.02^b^
	Control		6.65	+	3.80 ± 0.03^b^	4.09 ± 0.03^b^	4.53 ± 0.04^a^	7.21 ± 0.04^c^
6	GWE	20	6.80	±	3.77 ± 0.05^a^	4.20 ± 0.01^a^	3.78 ± 0.01^a^	8.11 ± 0.03^a^
		40	6.71	−	3.57 ± 0.06^a^	4.08 ± 0.02^a^	3.57 ± 0.03^a^	7.11 ± 0.007^b^
		60	6.53	−	2.67 ± 0.04^b^	3.33 ± 0.02^a^	3.25 ± 0.03^a^	6.99 ± 0.01^b^
	PWE	20	6.47	±	3.30 ± 0.01^a^	4.12 ± 0.01^a^	3.46 ± 0.01^a^	7.14 ± 0.04^b^
		40	6.67	−	3.34 ± 0.03^a^	4.98 ± 0.00^a^	3.34 ± 0.01^a^	6.96 ± 0.05^b^
		60	6.68	−	2.30 ± 0.06^b^	3.02 ± 0.04^a^	3.16 ± 0.02^a^	6.47 ± 0.00^b^
	Control		6.87	+	4.12 ± 0.03^c^	4.80 ± 0.03^b^	5.44 ± 0.03^b^	8.69 ± 0.03^c^
9	GWE	20	6.80	±	3.77 ± 0.05^a^	4.20 ± 0.01^a^	3.78 ± 0.01^a^	8.11 ± 0.03^a^
		40	6.71	−	3.57 ± 0.06^a^	4.08 ± 0.02^a^	3.57 ± 0.03^a^	7.11 ± 0.007^b^
		60	6.53	−	2.67 ± 0.04^b^	3.33 ± 0.02^a^	3.25 ± 0.03^a^	6.99 ± 0.01^b^
	PWE	20	6.47	±	3.30 ± 0.01^a^	4.12 ± 0.01^a^	3.46 ± 0.01^a^	7.14 ± 0.04^b^
		40	6.67	−	3.34 ± 0.03^a^	4.98 ± 0.00^a^	3.34 ± 0.01^a^	6.96 ± 0.05^b^
		60	6.68	−	2.30 ± 0.06^b^	3.02 ± 0.04^a^	3.16 ± 0.02^a^	6.47 ± 0.00^b^
	Control		6.87	+	4.12 ± 0.03^c^	4.80 ± 0.03^b^	5.44 ± 0.03^b^	8.69 ± 0.03^c^

Total bacterial count and coliform count on day 1 were 3.11 ± 0.15 and 1.7 ± 0.23 log CFU/g, respectively. The results of total bacterial counts showed significant differences between treatment and control samples (*p* < .05). No significant difference was observed in total counts of bacteria, coliforms, and *E. coli* in all treatments on day 1. However, with increasing the concentration of extracts, a decrease in the total count of microorganisms was observed. In the control and sample treated with 20 g of PWE and GWE, *S. Typhimurium* and in the sample with 20 g of GWE and control, *S. aureus* was isolated.

The results of *the E.coli* count showed no statistically significant difference between control and treatments (*p* > .05).

On the third day of storage, the control samples compared to the first day of treatment; an increase of 0.9 log CFU/g showed an overall microbial load rate (*p* < .05). It was 1.5 log CFU/g for 3 days, but the increase in total microbial load was not significant between treatments with different amounts of PWE and GWE (*p* > .05). Coliforms were observed in control and treatment samples (*p* < .05). However, this increase was higher among treatments, with the increase of both extracts, but no significant difference was observed between treatments and control. The results showed that the addition of pomegranate and grape extracts on the third day prevented the increasing of about 1.3 log CFU/g Pomegranate and grape extract did not affect *E. coli* and *S. Typhimurium* but the grape extract in 60 g and all amounts of pomegranate extracts on the third day killed Gram‐positive *S. aureus*. PWE and GWE significantly declined the number of coliforms in control samples (2.66 log CFU/g). An increase in the concentration of the extract resulted in a decrease in coliforms in the treated groups; this decrease was also significant (*p* > .05). The reason for the higher resistance of *E. coli* to *S. aureus* is the difference between the membrane structure of Gram‐positive and Gram‐negative bacteria and the difference in their peptidoglycan thickness. Gram‐positive bacteria such as *S. aureus* have multilayered and thick peptidoglycans, but Gram‐negative bacteria such as *E. coli* have thinner peptidoglycans but their outer membranes have lipopolysaccharides with little permeability. For this reason, the resistance of *E. coli* is greater than *S. Aureus*. (Feng et al., 2005; Kim et al., 2007).

On the sixth day of storage, control samples showed an increase of 4.2 log CFU/g compared to the first day (*p* < .05). Total microbial load in samples treated with different amounts of extracts were significantly higher than the first and third days (*p* < .05). However, after 6 days, treated carcasses had a significant decrease in total microbial counts than the control group, but this decrease among treatments in both extracts was not significant. A significant increase was observed in total coliform counts in the control and treated samples (*p* < .05). There was a significant decrease in coliforms but this decrease was not significant with increasing concentration of extracts (*p* > .05). PWE and 60 g of grape extract on day 6 killed *S. aureus* or inhibited bacterial growth.

The highest total counts of bacteria were observed in control on the ninth day of storage so that on this day the total microbial load increased to about 5 log cycles compared to day 1 (Table 3). The lowest microbial count after 9 days was in a sample containing 60 g of PWE. After 9 days of storage, a significant increase in total coliforms was observed in control samples compared to treatments (*p* < .05) but this increase in coliform counts was not significantly affected by increasing the concentration of extracts (*p* > .05). Increasing in extracts concentrations as well between them did not have a significant effect on the reduction of *E. coli*, *S. Typhimurium,* and *S. aureus*.

According to the Iranian national standard, the maximum acceptable microbial load in raw chicken meat is 10^5^ log CFU/g. Based on the finding of this research, the microbial count of the control sample after 3 days was close to the standard range but the application of extracts preserved the poultry carcasses close to the standard range until the sixth day of storage. Javadi and safarmashaei (2011) determined the microbiological quality of poultry meat marketed in the northwest of Iran (Tabriz). They showed that the mean aerobic plate count in broiler meat was 6.56–7.15 log CFU/g. *S. aureus* was isolated from 65% and coliforms in 100% of poultry meat samples. The prevalence of *Salmonella* was 0%.

Effect of washing broilers wings with 8% (w/v) *Rhus coriaria* L. extract and 2% (w/v) lactic acid solution was evaluated on microbial reduction immediately after 10 min of treatment. The reduction in total microbial bacteria was 2.6 and 1.7 log CFU/g, respectively, and the shelf life of the treated samples was 14 days compared to the 7 days shelf life of the control (distilled water). Samples treated with distilled water had a better color index.

The mixture of lemon sour, plum, and sour orange extract was applied to chicken skin inoculated with 10^5^ log CFU/g *Campylobacter* to evaluate their synergistic or antimicrobial antagonistic effects. After incubation (48 hr at 4°C), no Campylobacter was detected in samples treated with the mixture of extracts. Panelists found that the mixture of lemon sour and plum gave the chicken wings the best flavor. These extracts proposed as a natural antimicrobial that has the potential to replace or reduce *Campylobacter* (Valtierra‐Rodriguez, Heredia, Garcia, & Sanchez, 2010).

A comprehensive overview of the antimicrobial effects of a variety of physical, chemical, and synthetic methods on the decontamination of poultry carcasses and its components has been provided. Many studies have shown that chemical treatments are more effective than physical treatments. In general, the decrease in the bacterial load of chicken carcasses has been reported to be about 1–2.2 log cycles (Loretz et al., 2010).

Kanatt et al. (2010) showed that pomegranate peel extract was effective against Gram‐positive bacteria even at a concentration of 0.01%. Other researchers have shown that pomegranate fruit extract (at 1% concentration) inhibits *S. aureus* growth (Braga et al., 2005). However, in the case of Gram‐negative bacteria, pomegranate peel extract was effective on *Pseudomonas* spp. At 0.1% concentration and higher (up to 0.5%), this extract did not inhibit the growth of *E. coli* and *S. Typhimurium*. Reports also showed that pomegranate peel extract was less effective against *E. coli* and *Pseudomonas* (Negi, Jayaprakasha, & Jena, 2003). Other studies have also shown that most plant extracts are ineffective against Gram‐negative microorganisms (Oliveira et al., 2008). Natural herbal extracts contain a range of phytochemicals of phenolics, yet the exact mechanism of cellular damage due to complex processes is not clear and unknown. The mechanism of antimicrobial activity of extracts containing phenols is likely to be due to the disorder in the cell membrane.

Tavakoli et al. (2011) evaluated the effect of ozone on bacterial contamination of poultry carcasses at an industrial slaughterhouse in Tehran. They showed that the mean of total bacteria count in the control was 1.0^2^ × 10^5^ log CFU/g while in the treated groups with two exposure times of 4 and 10 min consisting of 4 ppm ozone were 9.14 × 10^4^ and 1.76 × 10^4^ log CFU/g, with 6 ppm of ozone were 3.51 × 10^4^ and 1.15 × 10^3^ log CFU/g and with 8 ppm of ozone were 5.32 × 10^2^ and 1.06 × 10^2^ Log CFU/g, respectively. There was no *Salmonella* in the samples treated with 8 ppm ozone with both exposure times of 4 and 10 min.

### 3.4 Total volatile nitrogen

In all samples, the trend of TVN was rising in the control and treatments. The increase in TVN in the treatment groups was at a slower rate than the control. TVN on the ninth day in treatment was approximately 3 times in control at the first day. The most effective treatment in preventing the reduction of volatile nitrogen compounds is the group treated with 60 g of pomegranate extract in 1 liter of chilled water after 9 days was 10 mg/100 g less than the control. This value was lower on day 6 in the effective treatment group containing 60 g of pomegranate extract was 7 mg/100 g less than the control. According to the instructions of Public Health's Bureau of the National Veterinary Office, poultry meat will not be consumed if the amount of TVN in poultry exceeds 27 mg/100 g. If the maximum is 20, between 21 and 24, and 25 to 27 mg/100 g, it will be desirable, usable, and use rapidly, respectively (IVO, 2005). Treating with PWE and GWE is well capable of maintaining chemical quality from the standpoint of VOC as a conventional indicator in assessing poultry carcasses for veterinary inspection until day 6 (Table 4). Based on the findings of this study, the treatment of poultry carcass with PWE and GWE as a natural preservative can extend the shelf life of fresh poultry by up to 6 days from the TVN index point of view. Bacterial activities and enzymes present in meat affect the amount of volatile nitrogenous bases (Razavi Shirzi, 2007). In general, there is a direct relationship between the production of volatile nitrogenous bases and bacterial growth (Gram & Huss, 1996). Badee, Moawd, ElNoketi, and Gouda (2013) found similar results in the study of the effects of Marjoram essential oil on the quality and shelf life of fresh poultry meat and showed that the amount of TVN in control samples increased more rapidly during storage, while the highest shelf life was related to the treated samples with higher doses of essential oil. Also, the results of the present study are in accordance with the results of Molaee Aghaee, Kamkar, and Akhondzadeh Basti (2016) in the effectiveness of biodegradable formulation with garlic essential oil on the chemical characteristics of chicken fillets. They found that at levels more than 1 and 2% of garlic essential oil no significant difference was found in TVN and their measured values were below 25 mg/100 g by the end of the study and the treated samples were still within the acceptable range. In another research, the effect of radiation and edible coatings containing ethanolic extract of papaya on enhancing shelf life of chicken was investigated. The mean of TVN on days 6 and 9 in the control group was 26.15 and 35.73 mg /100 g, respectively. However, this amount was 22.57 and 27.29 mg /100 g in the coated samples on these days, respectively (Abdeldaiem, 2015).

**TABLE 4:** Chemical analysis of poultry carcasses treated with pomegranate and grape waste extract (immersed 5 min in chiller)

Storage day	Extract/Chiller water (ml/L)		TVN (Mg/100g)
1	GWE	20	11.24 ± 0.23^a^
		40	11.38 ± 0.14^b^
		60	11.38 ± 0.22^b^
	PWE	20	11.34 ± 0.09^b^
		40	11.38 ± 0.10^b^
		60	11.27 ± 0.05^a^
	Control		11.30 ± 0.11^a^
3	GWE	20	18.84 ± 0.04^a^
		40	18.15 ± 0.11^b^
		60	16.74 ± 0.06^c^
	PWE	20	18.80 ± 0.05^a^
		40	18.15 ± 0.04^b^
		60	16.62 ± 0.11^c^
	Control		23.60 ± 0.09^d^
6	GWE	20	28.38 ± 0.03^a^
		40	25.12 ± 0.10^b^
		60	22.76 ± 0.04^c^
	PWE	20	28.20 ± 0.04^a^
		40	26.42 ± 0.03^d^
		60	23.04 ± 0.02^c^
	Control		33.80 ± 0.04^e^
9	GWE	20	28.38 ± 0.03^a^
		40	25.12 ± 0.10^b^
		60	22.76 ± 0.04^c^
	PWE	20	28.20 ± 0.04^a^
		40	26.42 ± 0.03^d^
		60	23.04 ± 0.02^c^
	Control		33.80 ± 0.04^e^

### 3.5 Sensory evaluation results

**FIGURE 2:**
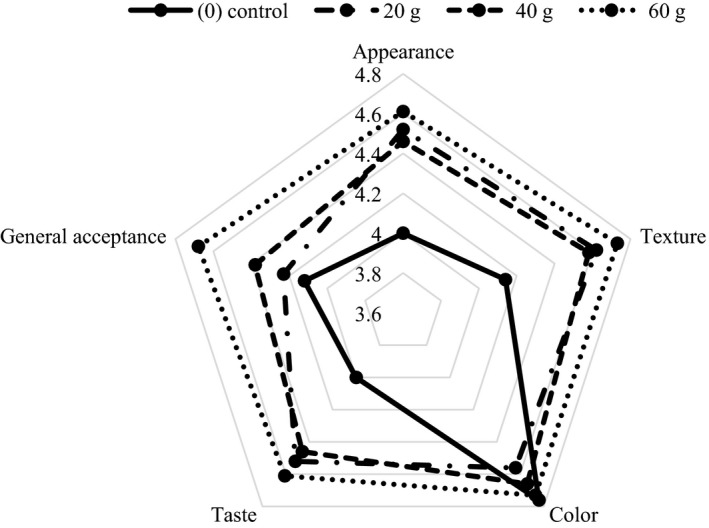
Sensory evaluation of poultry carcasses containing grape extract during storage at 6 days

The sensory evaluation in 6 days after storage is shown in Figures 1 and 2. A significant difference between different treatments and the control sample was observed (*p* < .05). It was observed that sensory properties (appearance, texture, color, taste, general acceptance) of treated samples with pomegranate and grape extracts after 6 days were better than controls. Results showed that addition of extracts to chicken carcass has been caused to increase the shelf life and preserve the quality of samples. The highest acceptance, in doses of pomegranate and grape extracts at 60 g, was observed. The results of sensory evaluation showed that the use of PW and GW extracts as a new industrial‐scale approach to reduce carcass contamination of slaughter poultry and the use of natural compounds as an antimicrobial compound were acceptable to consumers and had no adverse effect on organoleptic characteristics of treated carcasses and maintain the quality desired by panelists.

The effect of pomegranate peel extract on the shelf life of chicken products was investigated, which reported that adding pomegranate peel extract did not affect the sensory analysis of the samples. After 20 days of storage in cold conditions, it did have acceptable sensory properties (Kanatt et al., 2010).

## 4 CONCLUSION

The decontamination effects of natural antimicrobials derived from grape and pomegranate waste extracts on poultry carcasses (total bacteria, *Staphylococcus aureus, coliforms, Escherichia coli,* total volatile nitrogen, and sensory properties) in a slaughterhouse (chiller) were evaluated. It was observed that using recommended amounts of grape and pomegranate waste extracts can be significantly effective in decontamination and keeping poultry carcasses quality and improve the shelf life. After 9 days of storage time at 4°C, treated carcasses had a significant decrease (2.00–2.22 log CFU/g) in total microbial counts than the control. Increasing the applied concentration of PW and GW extract decreased the rate of TVN formation during storage in poultry carcasses.

Immersion poultry carcasses in the natural plant‐based extract‐like grape and pomegranate waste extracts in slaughterhouse chillers could inhibit bacterial growth and postpone chemical changes to extend shelf life. This decontamination procedure has no adverse effect on product appearance or any other sensory aspect and is a low‐cost applicable technique. This method will enable food processors to deliver larger amounts of high quality with extended shelf life and improvement of storage safety of chicken meat.

## CONFLICT OF INTEREST

None declared.
